# The Identification of *SQS*/*SQE*/*OSC* Gene Families in Regulating the Biosynthesis of Triterpenes in *Potentilla anserina*

**DOI:** 10.3390/molecules28062782

**Published:** 2023-03-20

**Authors:** Yangmiao Jiao, Xu Li, Xueshuang Huang, Fan Liu, Zaiqi Zhang, Liang Cao

**Affiliations:** 1 Hunan Provincial Key Laboratory of Dong Medicine, Ethnic Medicine Research Center, Hunan University of Medicine, Huaihua 418000, China; 13437276084@163.com (Y.J.); lx939308916@163.com (X.L.); xueshuanghuang@126.com (X.H.); liuqing@stu.hunau.edu.cn (F.L.); 2 Hunan Provincial Key Laboratory for Synthetic Biology of Traditional Chinese Medicine, School of Pharmacy, Hunan University of Medicine, Huaihua 418000, China

**Keywords:** *Potentilla anserina*, triterpenoids, squalene synthases, squalene epoxidases, oxidosqualene cyclases

## Abstract

The tuberous roots of *Potentilla anserina* (Pan) are an edible and medicinal resource in Qinghai–Tibetan Plateau, China. The triterpenoids from tuberous roots have shown promising anti-cancer, hepatoprotective, and anti-inflammatory properties. In this study, we carried out phylogenetic analysis of squalene synthases (*SQSs*), squalene epoxidases (*SQEs*), and oxidosqualene cyclases (*OSCs*) in the pathway of triterpenes. In total, 6, 26, and 20 genes of *SQSs*, *SQEs*, and *OSCs* were retrieved from the genome of Pan, respectively. Moreover, 6 *SQSs* and 25 *SQEs* genes expressed in two sub-genomes (A and B) of Pan. *SQSs* were not expanded after whole-genome duplication (WGD), and the duplicated genes were detected in *SQEs*. Twenty *OSCs* were divided into two clades of cycloartenol synthases (CASs) and *β*-amyrin synthases (*β*-ASs) by a phylogenetic tree, characterized with gene duplication and evolutionary divergence. We speculated that *β*-ASs and CASs may participate in triterpenes synthesis. The data presented act as valuable references for future studies on the triterpene synthetic pathway of Pan.

## 1. Introduction

Triterpenes are one of the largest groups of secondary metabolites from natural origins with various skeletons; many deployed promising anti-cancer and anti-oxidant activity and are used widely in the pharmaceutical industry, such as ursolic acid, oleanolic acid, and ginsenoside [1,2]. Triterpenoids discovered from plants are commonly generated in the pathway of farnesyl pyrophosphate (FPP). squalene synthases (*SQSs*) catalyze FPP to squalene, and squalene is then oxidized by squalene epoxidases (*SQEs*) to 2,3-oxidosqualene, which is further converted into different triterpene skeletons by different members of oxidosqualene cyclases (*OSCs*) (Figure S1). Triterpene scaffolds are further oxidized or glycosylated into different structures of triterpenoids by various cytochrome P450 monooxygenases (CYP450) and UDP glucosyltransferases or cellulose synthase-like M-subfamilies [3]. In previous studies, 121 triterpenes skeletons have been summarized according to different conformation and ring numbers, among which 51 skeletons have been experimentally characterized as products of *OSCs* [4]. Interestingly, 24 skeletons not reported from nature sources were generated by *OSCs* in heterologous expressions [4]. Protosteryl and dammarenyl cations were parents of many triterpene skeletal types. Protosteryl cation with chair–boat–chair (CBC) conformation was further catalyzed by the *OSCs* of cycloartenol synthases (CASs), lanosterol synthases (LASs), and cucurbitadienol synthases (CDSs) to form products of cycloartenol, lanosterol, and cucurbitadienol, respectively, which are associated with the tetracyclic triterpene skeleton (6-6-6-5). While dammarenyl cation derived from the chair–chair–chair (CCC) conformation was the parent for classes of pentacyclic triterpenes, such as *α*-amyrin (6-6-6-6-6), *β*-amyrin (6-6-6-6-6), and lupeol (6-6-6-6-5), which were catalyzed by *OSCs* of α-amyrin synthases (*α*-ASs), *β*-amyrin synthases (*β*-ASs), and lupeol synthases (LUSs), respectively [5,6]. The stabilization of intermediate cations, steric hindrance, and the conformation of active center amino acid residues are primary factors affecting triterpene formation. *OSCs* in plants are encoded by polygenic families, and *LASs*, *LUSs*, and *β-ASs* may be differentiated and evolved from *CASs* [5,6]. The lengths of most exons of *OSCs* are conservative, but the lengths of exons 4, 7, and 9 show diversity [7]. Taking advantage of abundant genome resources to explore the key enzymes in the synthetic pathway of natural products and conducting research on the biosynthesis of bioactive small compounds can benefit the modern healthcare system [8].

Tuberous roots of *Potentilla anserina* (Pan), also named silverweed cinquefoil roots, served as important food and medicine sources for Tibetans in the Qinghai–Tibetan Plateau, China, over thousands of years. It is an important source of starch and bioactive compounds of triterpenoids and flavonoids [9,10,11,12]. Interestingly, all triterpenoids reported from Pan were pentacyclic triterpenes, including oleanane, ursane, and lupine types [9,13,14], which displayed outstanding hepatoprotective and anti-inflammatory effects [9,13]. The structure–activity relationships disclosed that 3*α*-OH, 19*β*-CH_3_, 20*α*-CH_3_, 20*β*-CH_3_, 21*α*-OH, and 30-OH groups in the structure of triterpenoid or saponins could strengthen the hepatoprotective and anti-inflammatory activities [13]. Many medicinal plants in the genus of *Potentilla* have been used in traditional Chinese and ethnic medicine. For example, the whole plants of *P. chinensis* Ser. and *P. discolor* Bge. were recorded in the Chinese pharmacopeia [14,15]. Studying the triterpene synthesis pathway in Pan is of great significance.

Pan and *P. micrantha* (Pmi) are two species in the genus of *Potentilla* with genomes that have recently been reported. Both species have a close genetic relationship to the woodland strawberry *Fragaria vesca* (Fve). The genome of Pan contains 14 chromosomes, diverged from that of Pmi 28.52 million years ago (Mya). Furthermore, Pan has undergone a recent whole-genome duplication (WGD) to ensure tetraploidization. The sub-genome structures of Pan are characterized with the A sub-genome, which is larger than the B sub-genome and phylogenetically closer to the genome of Pmi. In general, the A sub-genome had higher homoeologous gene expression in the tuberous root [16]. Fifty-one percent of genes of Pan and Pmi are in a ratio of 2:1, based on the collinearity analysis of 26,032 genes [16]. In this study, we analyzed *SQSs*, *SQEs*, and *OSCs* involved in the biosynthesis of triterpenes in Pan and revealed function and evolutionary relationships via comparative genomic studies, which could facilitate research on bioactive triterpenes in Pan.

## 2. Results

### 2.1. The Identification and Characterization of SQS/SQE/OSC Gene Families in Pan

**Figure 1:**
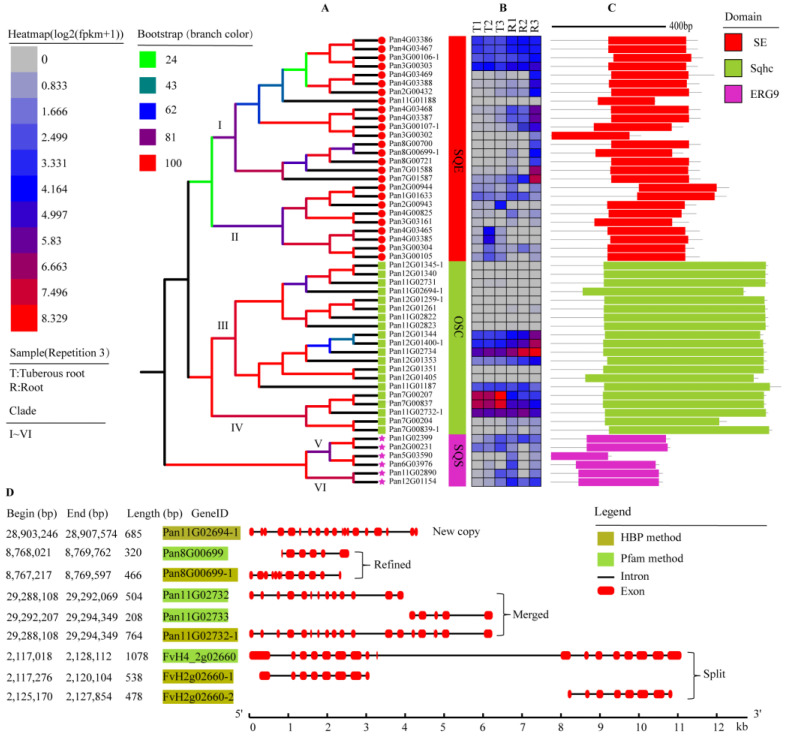
The phylogenetic relationship (**A**), gene expression (**B**), and conserved domain (**C**) of *SQSs*/*SQEs*/*OSCs* from *Potentilla anserina* (Pan). (**A**) The phylogenetic tree of *SQEs* (clades I and II), the phylogenetic tree of *OSCs* (clades III and IV), and the phylogenetic tree of *SQSs* (clades V and VI). (**B**) The expression level of *SQSs*/*SQEs*/*OSCs* in the tissue of tuberous root (T1–T3) and root (R1–R3) yields. (**C**) The conserved domains of *SQSs*/*SQEs*/*OSCs*. (**D**) The correction of the HBP method to Pfam analysis. New copy: a new copy of genes was obtained by the HBP method; Refined: genes were adjusted; Merged: multiple genes were merged into a complete gene; Split: one gene was split into multiple genes.

In this study, from the genomic data of Pan, we retrieved 6, 26, and 20 genes for *SQSs*, *SQEs*, and *OSCs*, respectively (Figure 1A and Table S2). Six copies of *SQSs* were obtained by searching the genomic data of Pan with the Protein family database (Pfam, ID PF00494), while zero copies were derived from Homolog-based prediction (HBP). For *SQEs*, 25 copies of *SQES* were acquired using Pfam (ID PF08491) for searching, while 24 were obtained by HBP; among them, 23 were intersected, 1 new copy (*Pan3G00107-1*) was annotated by HBP, and 2 were refined with incomplete annotation (*Pan8G00699-1* and *Pan3G00106-1*) (Figure 1D, Table S2). For *OSCs*, 18 and 19 copies were obtained via the Pfam database (IDs PF13249 and PF13243) and the HBP method, respectively, with 16 copies intersected. *Pan11G02732* and *Pan11G02733* mapped by Pfam were merged to *Pan11G02732-1* by HBP (Figure 1D). *Pan12G01400* (annotated) and *Pan12G01401* (not acquired by Pfam) were merged to *Pan12G01400-1* by the HBP method; moreover, the HBP method refined two genes (*Pan7G00839-1* and *Pan12G01259-1*) and screened out one new copy (*Pan11G02694-1*) of *OSCs* (Table S2).

According to the Conserved Domain Database (CDD), we obtained the domains of *SQSs*/*SQEs*/*OSCs* (Figure 1C). The amino acid lengths of predicted products from *SQSs*, *SQEs*, and *OSCs* ranged from 318 bp to 627 bp, 215 bp to 420 bp, and 619 bp to 778 bp, respectively, and the isoelectric point ranged from 7.11 to 9.32, 5.01 to 8.84, and 5.73 to 7.61, respectively. Subcellular localization revealed that *SQSs* were located in organelle membranes and chloroplasts, products of *SQEs* located in endomembrane system, and organelle membranes, while proteins coded by *OSCs* were mainly located in plasma membranes and the nucleus (Table S2). In total, 6 *SQSs* genes and 25 *SQEs* genes were detected with different expression levels in root and tuberous root, respectively, and 8 *OSCs* genes were expressed in root and tuberous root (Figure 1B).

### 2.2. The Chromosomal Locations and Collinearity Analysis of SQSs/SQEs/OSCs

**Figure 2:**
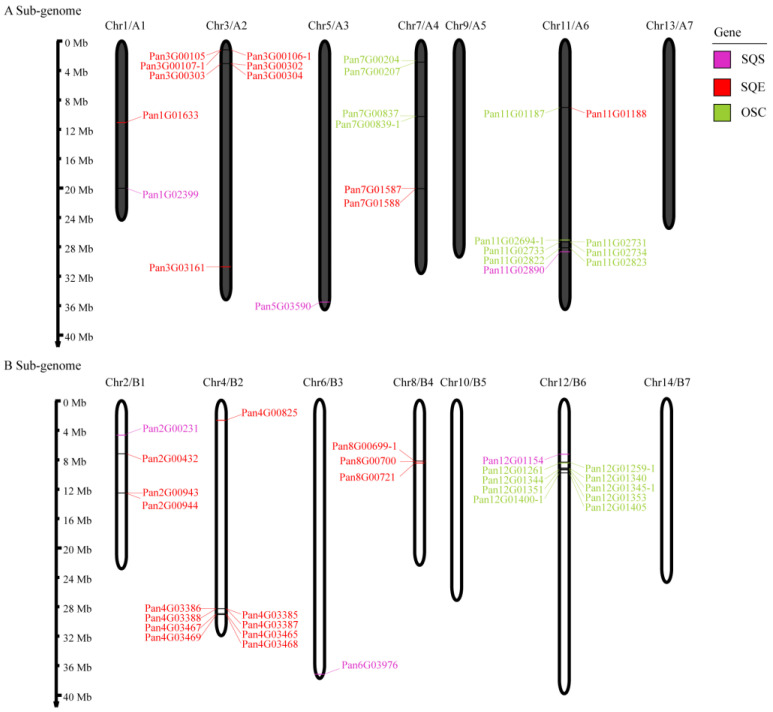
*SQS*/*SQE*/*OSC* locations on the *Potentilla anserina* (Pan) chromosomes. A Sub-genome (up, A1–A7), B sub-genome (bottom, B1–B7).

The genome of Pan contains 14 chromosomes, which underwent a tetraploidization in 6.4 Mya. The A sub-genome (A1–A7, from Chr1 to Chr13, odd numbers) and B sub-genome (B1–B7, from Chr2 to Chr14, even numbers) showed good collinearity [16]. *SQSs* were symmetrically distributed on two sub-genomes. *SQEs* were scattered on seven chromosomes such as Chr1 and Chr2, and the number of products located on the B sub-genome was larger than that of the A sub-genome, and there were tandem-duplicated genes on Chr2, Chr3, Chr4, Chr7, and Chr8. *OSCs* were concentrated and distributed on Chr7, Chr11, and Chr12 with tandem-duplicated genes, with the A sub-genome containing more copies (Figure 2).

### 2.3. The Phylogenetic Analysis of SQSs/SQEs

**Figure 3:**
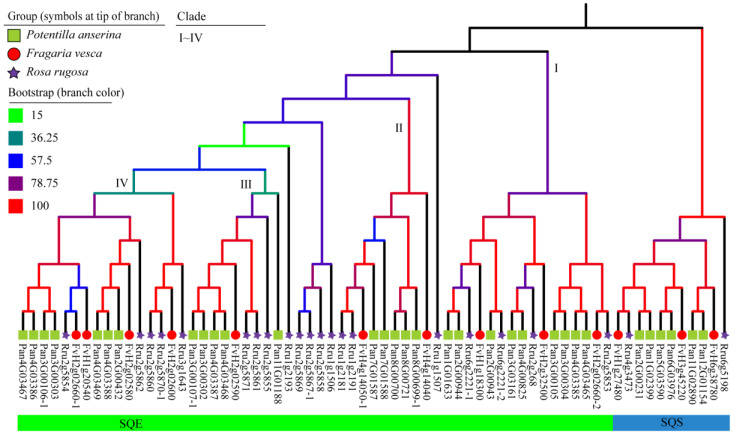
The phylogenetic tree of *SQSs* and *SQEs*. *SQSs* and *SQEs* of *Potentilla anserina* (Pan), *Fragaria vesca* (Fve), and *Rosa rugosa* (Rru) were included in the phylogenetic tree. Clade I to clade IV included genes of *SQEs*; the right clade was *SQSs*.

We constructed a phylogenetic tree of *SQSs* and *SQEs* of Pan, Fve, and *Rosa rugosa* (Rru) (Figure 3, Table S3). Orthologous genes showed good collinearity, i.e., in *SQSs*, the Pan:Fve:Rru ratio was 6:3:2, and *FvH1g27480*, *Rru4g3473*, *Pan1G02399*, and *Pan2G00231* were distributed on a phylogenetic tree branch. *FvH3g45220*, *Pan5G03590*, and *Pan6G03976* were grouped together, and *FvH6g38780*, *Pan11G02890*, and *Pan12G01154* were grouped together. In *SQEs*, the Pan:Fve:Rru ratio was 26:10:20, and Pan was found to have many tandem-duplicated genes, such as in Chr3 (*Pan3G00105* and *Pan3G00304*) and Chr4 (*Pan4G03465* and *Pan4G03385*) in clade I (Figure 3). Meanwhile, in other branches, Rru was found to have a unique gene doubling event.

### 2.4. Phylogenetic Analysis of OSCs

**Figure 4:**
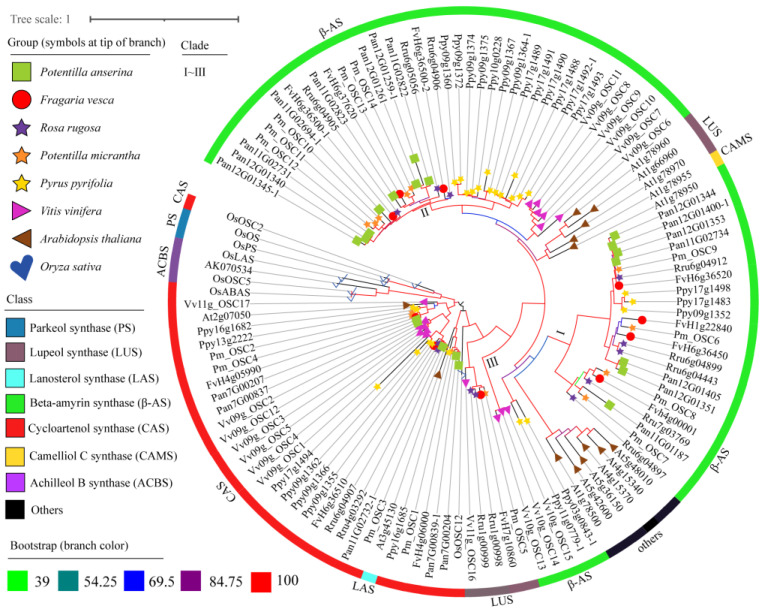
Phylogenetic tree of *OSCs*. *OSCs* of *Potentilla anserina* (Pan), *Potentilla micrantha* (Pmi), *Fragaria vesca* (Fve), *Rosa rugosa* (Rru), *Pyrus pyrifolia* (Ppy), *Vitis vinifera* (Vvi), *Arabidopsis thaliana* (Ath), and *Oryza sativa* (Osa) were included in the phylogenetic tree. PS: parkeol synthase, LUS: lupeol synthase, LAS: lanosterol synthase, *β*-AS: beta-amyrin synthase, CAS: cycloartenol synthase, CAMS: camelliol C synthase, ACBS: achilleol B synthase.

The phylogenetic tree of *OSCs* retrieved from Pan, Fve, Rru, Pmi, *Pyrus pyrifolia* (Ppy), and *Vitis vinifera* (Vvi) was constructed along with the published/verified *OSCs* of *Arabidopsis thaliana* (Ath) and *Oryza sativa* (Osa) as outgroups (Figure 4; Tables S1 and S3). According to the functional annotation of KEGG and Swissport, products of *OSCs* were classified into eight categories (Figure 4). *OSCs* from Vvi, Fve, Rru, Ppy, and Pmi were classified into the CAS, LUS, and *β*-AS clades. *OSCs* from Pan were divided into two branches of CAS and *β*-AS. *CASs* were entirely distributed on the A sub-genome and divided into three sub-clades of *Pan7G00204* and *Pan7G00839-1*, *Pan7G00207* and *Pan7G00837*, and *Pan11G02732-1*, and the first two sub-clades on chromosome 7 (A4) had tandem duplication events. Meanwhile, *β-ASs* located on chromosomes 11 (A6) and 12 (B6) were divided into two branches, as shown in Figure 4 (clades I and II), characterized by a large number of tandem-duplicated genes.

## 3. Discussion

### 3.1. Gene Families Identified by the Pfam Database and the HBP Method

The development of sequencing technology and sophisticated genome assembly methods promotes the release of more genomes of plants. Generally, the reliability of subsequent genome analysis depends on the quality of assembly and the integrity of annotation. In this study, we used high-threshold Pfam results, along with the HBP method as a supplement, to identify genes in the pathway of triterpenes. We found that the HBP method had a correct rate (new copy and genes refined, merged, and split by HBP/all gene numbers) of 14–17% for *SQS*, *SQE*, and *OSC* gene families in different species (Table 1 and Table S3). There were merged *OSCs* in Pan and Ppy, according to the results (Table S3), possibly due to incomplete or low-accuracy annotation leading to fragmentation, or the wide existence of variable splicing in the genome. The split genes that appeared in Fve (i.e., FvH4_2g02660) and Rru were confirmed by the transcriptome data (Figure 1D, Table S4), which may be caused by tandem-duplicated genes, resulting in overly long annotation. The sequence of *Pan12G01345-1* obtained from the HBP method contained a premature termination codon which may be introduced during evolution.

Eleven *SQSs* were obtained via a search in the Pfam database (Figure S2), of which three *SQS*-like genes (*Pan4G02790*, *Pan3G00908*, and *Pan3G00874*) were located in independent branches with incomplete motifs. The other two *SQSs* (*Pan9G01625* and *Pan10G01518*) had complete PF00494 alignment information, but no corresponding motif, and KEGG annotation information. A possible explanation is that *SQSs* and the phytoene synthase family (*PSYs*) have structural similarities and share three conservative regions [17]. Thus, we included the remaining six *SQSs* in this study. In HBP analysis, only three *SQS*-like genes (*Pan4G02790*, *Pan3G00908*, and *Pan3G00874*) were obtained (Figure S2), probably because there were few reference genes for HBP. Therefore, accurate gene sets for homologous prediction and reliable databases for functional verification are critical in HBP analysis.

### 3.2. SQS/SQE/OSC Gene Family Expression and Evolution

Gene duplications were recognized as contributors to the evolution of genes with divergence functions. Sub-functional, hypofunctional, and neo-functional genes, as well as compensatory drift and neutral variation, may result from gene duplications [18]. Multiple paralogous genes may be generated by whole-genome polyploidization, segmental duplication, or tandem duplication [18]. The divergence of Vitales and Rosales occurred at about 121.9 Mya, while the Rosoideae divergence from Amygdaloideae occurred at 84.4 Mya [19]. The *Potentilla* genus was separated from *Fragaria* genus at about 40.68 Mya, followed by the divergence between Pan and Pmi (28.52 Mya) [16]. Pmi and Fve diverged at about 34.5 Mya [19], and Fve diverged from Rru at about 52.3 Mya [19]. An ancient genome-wide tripling event occurred in Vvi [20]. Plants in Rosaceae have experienced an ancient genome triplication, though no evidence of large-scale genome replication was found in Fve. It is speculated that chromosome rearrangement and genome shrinkage (or the selective loss of replication genes) cover up the ancient triplication event in Fve [21]. Rru has no additional genome-wide duplication after whole-genome triplication (WGT) [22]. The genome of Pan underwent a recent WGD event to form an allotetraploid after the divergence of Rosaceae, and the A sub-genome and the B sub-genome were found to have good collinearity [16].

According to the *OSC* phylogenetic trees and the distribution of *OSCs* on chromosomes, we discussed the characterization of *OSCs*. On the CAS clade, the Fve:Rru:Pan ratio was 3:2:5, and all *CASs* of Pan were distributed on the A sub-genome (Figure 4). Four copies of *OSCs* (*Pan7G00207*, *Pan7G00837*, *Pan7G00204*, and *Pan7G00839-1*) in chr7 were distributed into two branches, indicating that a tandem duplication event had taken place. The copies mentioned in chr7 showed a discrepant expression in root or tuberous root yields, and we speculated that the low expression may result from the shortening of coding sequences, resulting in sub-functionalization (Table S2).

In clade I of *β*-AS (*Rru6g04897*~*Pan12G01344*) (Figure 4), we hypothesized that *β*-AS has three ancestral copies, based on three genes of Vvi in the outgroup (clade III). The ratio of Fve:Rru:Pan was 4:5:7, and it was found that Pan has five *OSC* copies in chr12 and two copies in chr11. *Pan12G01344*, *Pan12G01400-1*, and *Pan12G01353* showed similar transcription levels (Table S2), and two copies of them may have expanded due to tandem duplication. In clade II of *β*-AS (*Rru6g04906*~*Pan12G01345-1*), the ratio of Fve:Rru:Pan was 3:3:8. The ratio of the sub-branch (*Rru6g05056*~*Pan11G02823*) of Fve:Rru:Pan was 1:1:4. Furthermore, the *OSCs* of Pan (*Pan11G02822*, *Pan11G02823*, *Pan12G01259-1*, and *Pan12G01261*) were evenly distributed in the A and B sub-genomes, and the genes generated by tandem duplication were not uniformly expressed in the tuberous root (Table S2).

Similarly, according to the phylogenetic tree of *SQSs* and *SQEs* (Figure 3), Fve and Pan had three and six *SQSs*, respectively. The *SQSs* of Pan distributed on the A and B sub-genomes symmetrically may result from the WGD event of Pan. For *SQEs*, one of *Pan2G00943* and *Pan2G00944* seemed to expand in clade I, while tandem duplication occurred in *Pan3G00304* or *Pan3G00105* and *Pan4G03465* or *Pan4G03385*, and the expression of duplicated genes was consistent in transcriptome data of root (Table S2). In clade II, *Pan8G00700*, *Pan8G00721*, and *Pan8G00699-1* were duplicated genes, and *Pan8G00699-1* had a higher expression in transcriptome data of root. In clade III, *Pan11G01188* had no expression in root and tuberous root yields, which may be attributed to spatio-temporal specific expression (Figure 3, Table S2).

### 3.3. The Functional Prediction of OCSs and Triterpene Synthesis in Pan

In the phylogenetic tree of eight species of *OSCs*, *CASs* were located in the outermost branch, which is in agreement with previous findings that *LASs*, *LUSs*, and *β-ASs* evolved from ancient *CASs* [5,6]. The classification of enzymes has been extensively studied with Osa as the outgroup [4]. *OsOSC2* converts 2,3-oxidosqualene to cycloartenol [23] when using the CBC conformation as the precursor under the catalyzation of S-adenosyl-L-methionine-sterol-C24-methyltransferase 1 (SMT1) and CYP450 [24], and cycloartenol could be converted into phytosterols [25] and cholesterol [26]. Moreover, steroidal diosgenin was obtained with further hydroxylation and cyclization [27]. OsPS (japonica subspecies) is a tetracyclic parkeol synthase. In contrast, OsOS (indica subspecies) synthesizes a novel pentacyclic triterpene orysatinol and 12 other triterpenes, and key amino acid residues were found to determine the functional divergence between OsPS and OsOS [28]. OsABAS is a multifunctional enzyme annotated as the achilleol B synthase, showing functions of both *α*-AS and *β*-AS enzymes [29]. The *OSCs* of Pan have been classified into two groups of CAS and *β*-AS. CASs synthesize the tetracyclic triterpene skeleton and may be involved in the synthesis of sterols. *SQEs* act as a rate-limiting enzyme in the steroid biosynthesis pathway [30]. The *β*-ASs detected in Pan are pentacyclic triterpene (oleanane) synthetases, which is consistent with a lot of oleanane-type triterpenoids isolated from Pan [10,14,15].

## 4. Materials and Methods

### 4.1. SQS/SQE/OSC Gene Family Identification

The sequenced and annotated genome data of *Potentilla anserina* (Pan) [16], *Potentilla micrantha* (Pmi) [31], *Fragaria vesca* (Fve) [32], *Rosa rugosa* (Rru) [22], and *Pyrus pyrifolia* (Ppy) [33] of Rosaceae were requested from the Genome Database for Rosaceae (GDR) (https://www.rosaceae.org/tools/jbrowse, accessed on 14 March 2023), while the sequence of *Vitis vinifera* (Vvi) [20] was downloaded from the National Center for Biotechnology Information (NCBI) database. *Oryza sativa* (Osa) and *Arabidopsis thaliana* (Ath), serving as outgroups, were also downloaded from the NCBI. PF13249 and PF13243 downloaded from Pfam (http://pfam.xfam.org/, accessed on 14 March 2023) were applied to search for *OSC* coding proteins using the hidden Markov model (HMM) (E-value < 1 × 10^−20^). In order to reduce the deviation caused by different gene annotation methods on a variety of genomes, we used homolog-based prediction (HBP) to predict the number of genes. The strategy of HBP is as follows. Firstly, the *OSC* (Table S1) protein sequences downloaded from the NCBI were aligned to the genome using TBLASTN with an E-value < 1 × 10^−5^. Secondly, the conjoined high-scoring pairs (HSPs) by Solar (https://github.com/gigascience/papers/tree/master/zhou2013/MTannotationBGI/solar, accessed on 14 March 2023) were applied to determine the rough genomic region for each gene. Thirdly, we extracted and extended 2 kb both upstream and downstream of the Solar region, and defined gene models using GeneWise (v2.4.1) [34]. We also filtered the predictions with less than 30% coverage. The functional annotation of resulted genes was actualized by the Pfam, KEGG (https://www.genome.jp/kegg/, accessed on 14 March 2023), and Swissprot (http://www.ebi.ac.uk/sprot, accessed on 14 March 2023) databases. The motif was recognized using Meme (https://meme-suite.org/meme/tools/meme, accessed on 14 March 2023), with a default number of 6. Furthermore, the domain was predicted under the CDD (https://www.ncbi.nlm.nih.gov/cdd/, accessed on 14 March 2023) database. We combined the two prediction results of Pfam and HBP, and then retained the accurate ones. The identification of *SQSs* (PF00494) and *SQEs* (PF08491) was realized using the same method (Table S1).

### 4.2. Physicochemical Properties of OSCs/SQEs/SQSs from Pan

Various physicochemical properties, including the number of amino acids, the molecular weight, the isoelectric point, the grand average of hydropathicity (GRAVY), and subcellular localization (Table S2), were analyzed. ExPASy (http://expasy.org/, accessed on 14 March 2023) was used to predict the molecular weight and isoelectric points. GRAVY was calculated using the GRAVY calculator (http://www.gravy-calculator.de/, accessed on 14 March 2023), and the subcellular localization was predicted using BUSCA (http://busca.biocomp.unibo.it/, accessed on 14 March 2023) [35].

### 4.3. Sequence Alignment and Phylogenetic Analysis

Protein sequence alignments were conducted by muscle5.1 [36]. The phylogenetic tree was established using iqtree1.5.5 [37] with the MFP model and 1000 replicates of bootstrap, and was displayed by itol (https://itol.embl.de/upload.cgi, accessed on 14 March 2023).

### 4.4. RNA-seq Analysis

The transcriptome data of Pan in root (SRR12053561, SRR12053562, and SRR12053563) and tuberous root (SRR12053557, SRR12053559, and SRR12053560) yields were analyzed by the HISAT2 + StringTie + Ballgown [38] pipeline. Meanwhile, the transcriptome data of Fve (SRR19907785, SRR19907789, SRR19907792, ERR9861197, ERR9861198, and ERR9861199) and Rru (SRR20883375, SRR20883380, and SRR20883382) were analyzed using the same method.

## 5. Conclusions

Triterpenes are major pharmacodynamic substances of Pan, which show good anti-inflammatory and hepatoprotective activity. In this study, we analyzed members of *OSC*, *SQS*, and *SQE* gene families in the triterpene synthesis pathway using Pfam and HBP methods. The results provide a research basis for understanding the biosynthetic pathway of triterpenes in Pan by clarifying the function and evolutionary relationship of triterpene-synthesis-related genes.

## Figures and Tables

**Table 1:** HBP predictions of *SQSs*, *SQEs*, and *OSCs* in four species.

Resource ^a^	Pan	Fve	Rru	Ppy
Total gene numbers	52	21	20	24
New copies	3	1	0	0
Refined genes	3	0	2	2
Merged genes	3	0	0	2
Split genes	0	2	1	0
Correct rates	17.3%	14.2%	15.0%	16.6%

^a^ The data in Table 1 are summarized from Table S3.

## Data Availability

The datasets analyzed in the current study are available in public databases of GDR and NCBI (https://www.rosaceae.org/ and https://www.ncbi.nlm.nih.gov/, accessed on 14 March 2023). The genomes of *Potentilla anserina* from https://figshare.com/projects/The_genome_assembly_and_annotation_files_of_Potentilla_anserina/83771 (accessed on 25 November 2021), *Potentilla micrantha* from https://www.rosaceae.org/rosaceae_downloads/Potentilla_micrantha/Pmicrantha-draft.v1.0/assembly/Potentilla_draft_genome.fasta.gz (accessed on 4 May 2018), *Fragaria vesca* from https://www.rosaceae.org/rosaceae_downloads/Fragaria_vesca/Fvesca-genome.v4.0.a1/assembly/Fragaria_vesca_v4.0.a1.fasta.gz (accessed on 1 January 2018), *Rosa rugosa* from https://www.rosaceae.org/rosaceae_downloads/Rosa_rugosa/Rosa_rugosa_v1.0/assembly/Rosa_rugosa_genome.fasta.gz (accessed on 31 August 2021), *Pyrus pyrifolia* from https://www.rosaceae.org/rosaceae_downloads/Pyrus_pyrifolia/ppyrifolia_v1.0/assembly/PPY_r1.0.pmol.fasta.gz (accessed on 11 January 2021), and *Vitis vinifera* from https://ftp.ncbi.nlm.nih.gov/genomes/all/GCF/000/003/745/GCF_000003745.3_12X/GCF_000003745.3_12X_genomic.fna.gz (accessed on 7 November 2019). The transcriptome data of *Potentilla anserina* (SRR12053557, SRR12053559, SRR12053560, SRR12053561, SRR12053562, and SRR12053563) (accessed on 19 June 2020), *Fragaria vesca* (SRR19907785, SRR19907789, SRR19907792) (accessed on 3 August 2022), (ERR9861197, ERR9861198, and ERR9861199) (accessed on 20 June 2022), and *Rosa rugosa* (SRR20883375, SRR20883380, and SRR20883382) (accessed on 14 September 2022) are download from https://www.ncbi.nlm.nih.gov/sra (accessed on 14 March 2023). All functional genes of different species are listed in Table S1. The origins of all genomes and transcriptomes are listed in Table S5.

## Supplementary Materials

References [39,40,41,42,43,44,45,46,47,48,49,50,51,52,53,54,55,56] are cited in Table S1. The following supporting information can be downloaded at: https://www.mdpi.com/article/10.3390/molecules28062782/s1. Figure S1: The biosynthetic pathway of triterpenes, Figure S2: The positions of five *SQS* genes inappropriately annotated by Pfam in the evolutionary tree. Supplementary Tables S1–S6: The supporting information of *OSC*/*SQE*/*SQS* gene families analyzed in this study. Table S1: Functional genes of *SQSs*/*SQEs*/*OSCs* in different species. Table S2: Expression and physicochemical properties of *SQSs*/*SQEs*/*OSCs* in Pan. Table S3: *SQSs*/*SQEs*/*OSCs* annotated by HBP and Pfam in different species. Table S4: Analysis of *FvH6g36500*/*FvH2g02660*/*Rru6g2221* structure and alternative splicing by RNA-seq. Table S5: NCBI under accession number for transcriptomes and genome resource of different species. Table S6: Protein sequences of *OSCs*/*SQSs*/*SQEs* in different species.
