# Supplementary material for: The Identification of SQS/SQE/OSC Gene Families in Regulating the Biosynthesis of Triterpenes in Potentilla anserina
Source: Molecules. 2023 Mar 20;28(6):2782. doi: 10.3390/molecules28062782 (PMC10051230; doi:10.3390/molecules28062782)
Supplement: Supplementary file 1 [file molecules-28-02782-s001.zip › Supplementary Files/Supplementary Figure.docx]

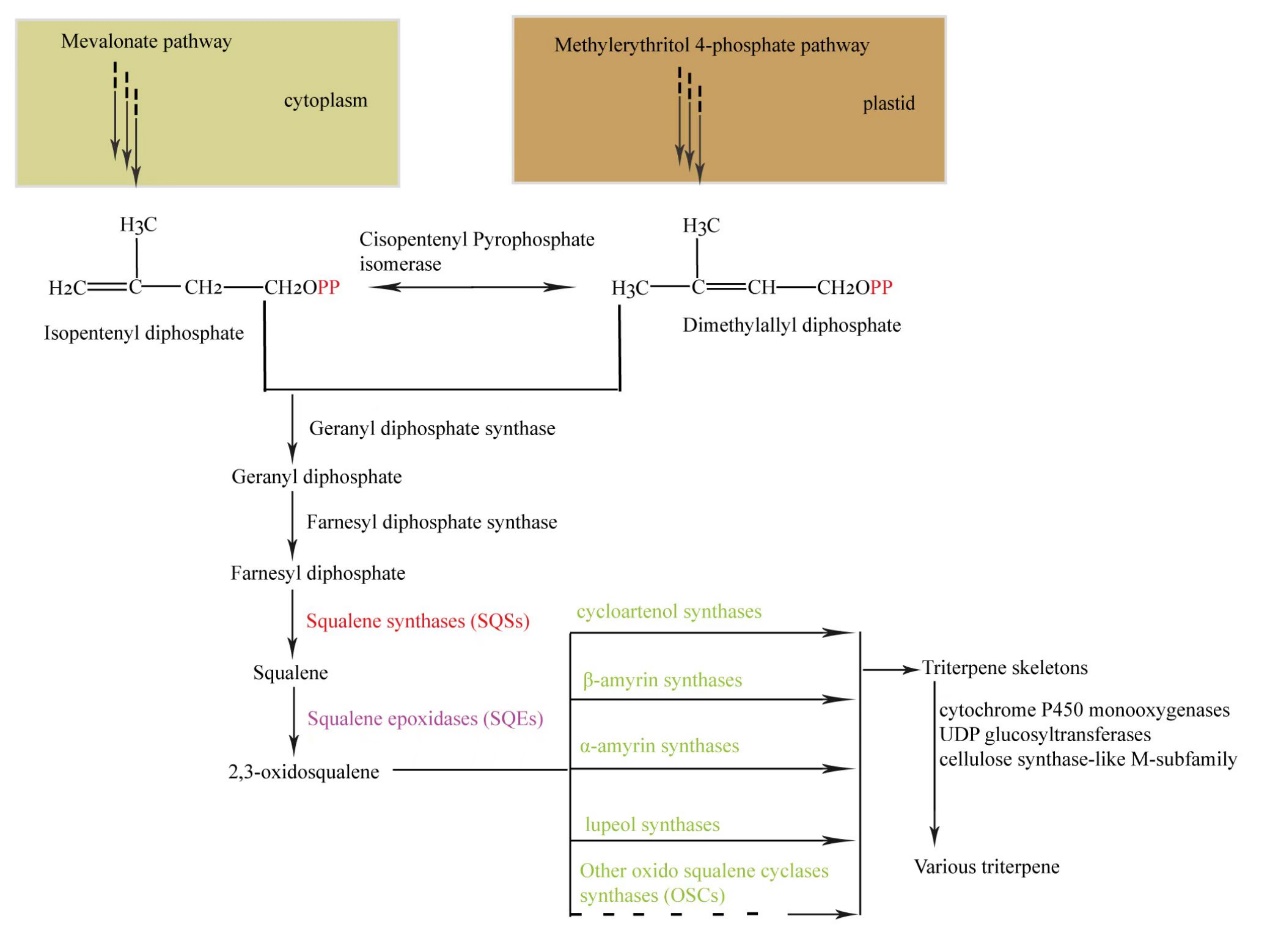
**Figure S1.** Biosynthetic pathway of triterpenes


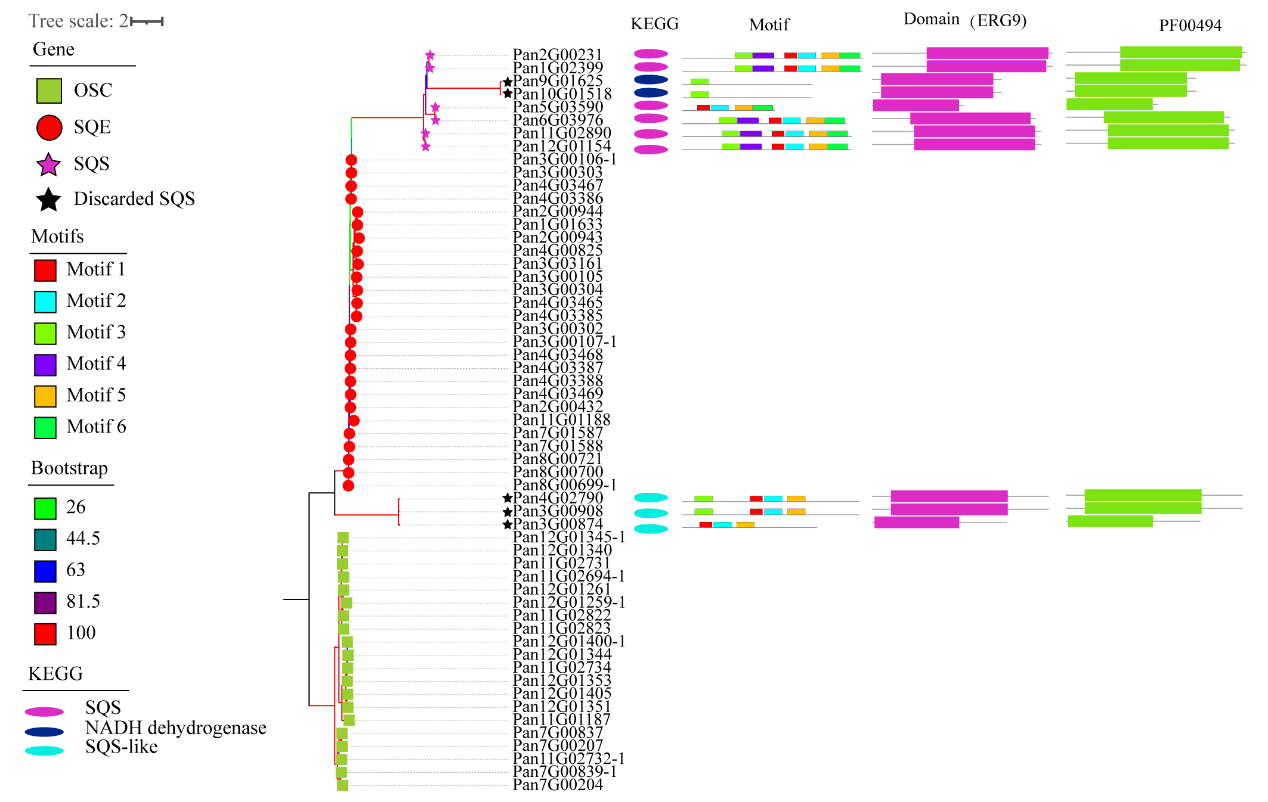


**Figure S2.** The positions of five *SQS*s inappropriately annotated by Pfam in the evolutionary tree.
